# Posture analysis during tooth extraction

**DOI:** 10.1038/s41405-025-00311-1

**Published:** 2025-02-17

**Authors:** Takashi Fukushima, Keisuke Sugahara, Kazuhiro Ito, Masahide Koyachi, Akihiro Nishiyama, Chihiro Kurihara, Shintaro Nakajima, Satoru Matsunaga, Akira Katakura

**Affiliations:** 1 Technical University of Munich, Chair of Performance Analysis and Sports Informatics, Georg-Brauchle-Ring 60/62, 80992 München, Germany; 2 Tokyo Gakugei University, Education Incubation Center, 4-1-1 Nukuikitamachi, Koganei, Tokyo 184-8501 Japan; 3 Tokyo Dental College, Department of Oral Pathobiological Science and Surgery, Tokyo Dental College, Tokyo, Japan 2-9-18, Kanda Misaki-cho, Chiyoda-ku, Tokyo Japan; 4 Tokyo Dental College, Department of Anatomy, Tokyo Dental College 2-9-18, Kanda Misaki-cho, Chiyoda-ku, Tokyo 101-0061 Japan

**Keywords:** Dental clinical teaching, Extended skills training in dentistry

## Abstract

**Introduction:**

Tooth extraction is one of the clinical internship requirements in Japan. Human posture during tooth extraction is important since poor posture can cause failure of the safe operation and musculoskeletal injuries. Only a few studies aimed to evaluate the posture, but most of them used manual or complex measurement methods which can lead to some inconvenient problems such as subjective biases, quantitativeness, and device availability. Thanks to the recent advancement of computer vision and technology, pose estimation has been widely used for kinematic analysis. However, none of the research has been used for posture analysis during tooth extraction.

**Aim:**

Therefore, this research aims to analyze posture kinematics during tooth extraction using pose estimation and find key kinematic variables for tooth extraction.

**Method:**

All participants were grouped into three; dental students, young dentists, and experienced dentists. They were asked to perform tooth extraction on a tooth extraction simulator while being video recorded. Pose estimation was used to extract joint locations on recorded videos. Joint angles of interest were calculated based on the extracted joint locations.

**Results:**

Right shoulder angles were significantly lower in the experienced dentists’ group than in other groups,

**Discussion:**

Which has been pointed out by other research as a crucial point.

**Conclusion:**

Although sample size is a main concern in this study, the result shows that pose estimation can be useful in posture analysis during tooth extraction.

## Introduction

### Tooth extraction

Since clinical internships became a requirement for dentists in 2006 in Japan, dentistry students have had fewer opportunities to practice invasive procedures such as tooth extraction on actual patients during their training. To address this issue, several dentistry schools use models fitted to mannequins; However, practice with a modeled tooth is limited to learning the technique and does not allow to experience and learn the intricacies associated with an actual tooth extraction. A simulator has been constructed to measure the force and direction of tooth extraction [1]. However, it should be noted that the posture of the surgeon is also of great importance in this procedure. This report aimed to analyze the utility of the simulator in teaching dentistry students and young dentists.

### Pose estimation

Due to recent advancements of computer vision, pose estimation is a hot topic in research. Pose estimation is a computer vision technique to estimate human joint locations from an image using a pre-trained machine learning model. The technique is used in a variety of fields such as kinematic analysis by Theia (Theia Markerless, Kingston, Canada), action recognition [2], and AI personal trainer tools by Kemtai (Kemtai, Petah Tikva, Israel). This technique contains a huge potential to replace a traditional motion capture system such as VICON (VICON Motion Systems Ltd., Oxford, UK). The problem of the traditional system is accessibility, affordability, and simplicity. Since the system requires multiple infrared cameras which are sensitive to sunlight, the motion capture needs to be in the laboratory [3, 4]. The system itself is expensive [5, 6]. Also, the system calibration and reflection markers’ placement are complicated [7].

### Posture during the tooth extraction

Posture during tooth extraction is important. Proper posture does not only help with safe surgical operation, but also it can prevent musculoskeletal disorders which are commonly experienced by dental health workers [8–10]. One research conducted posture analysis and evaluation, but they used manual measurement by using video recordings [11]. One problem of the evaluation is that the measurement can be subjective. Depending on the point that the researchers refer to, the measurement results can be changed. Another problem is that it is time-consuming to process many samples. To gain statistically meaningful results, a sample size needs to be big enough. Another study used an inertial measurement unit (IMU) to capture motions for kinematic analysis of dental posture [12]. The IMU can gain posture data quantitatively and easily by attaching the IMU sensors to the body. However, the device is often expensive; therefore, researchers or practitioners may not be able to afford the system. Also, the device is influenced by magnetic force since it contains a magnetometer, which would additionally cause negative effects on dental devices surrounding the IMU. Pose estimation can be a solution to overcome these problems.

### Research aim

Since the surgeon’s posture during the tooth extraction is important, a motion capture system is needed to conduct the kinematic analysis of the posture. However, traditional motion capture systems are not user-friendly for the aforementioned reasons. Therefore, this research aims to analyze posture kinematics during tooth extraction using pose estimation and find key kinematic variables for tooth extraction.

## Method

### Participant

In total, twelve participants were invited to this study. They were categorized as dentistry students, young dentists, experienced dentists based on their certificate levels in dental fields. The groups were called a group 1, 2, and 3, respectively. There were four dentistry students, six young dentists, and two experienced dentists.

### Set up

**Fig. 1:**
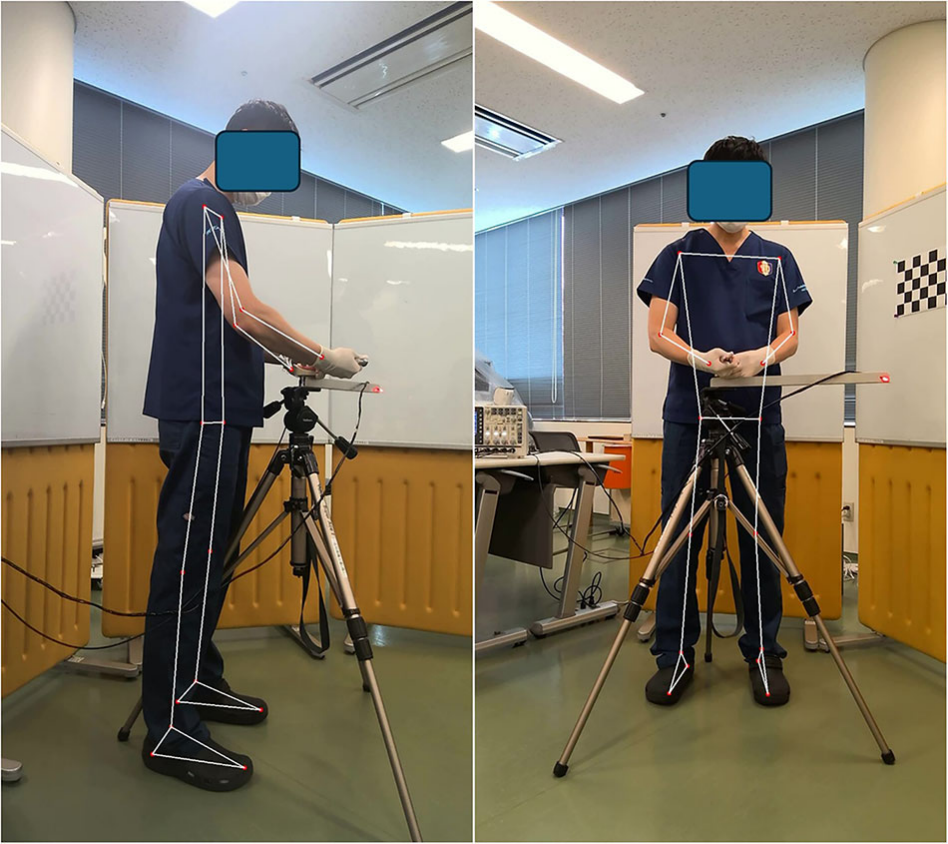
Front and side views of the participant and tooth extraction simulator with the LED light turned on.

In total, two Microsoft Surface Pro 8 tablets (Micorosoft, Redmond, WA) were placed in front of and lateral to each participant. The tooth extraction simulator was placed in front of the participant. The two cameras were timely synchronized using a LED light from the tooth extraction simulator. Figure 1 shows front and side views of the participant and tooth extraction simulator with the LED light turned on. The LED turns on when a subject applies force to the tooth extraction simulator. To reconstruct 3-dimensional (3D) data, both cameras were calibrated using Zhang’s method with an A4-sized chessboard which consists of five rows and seven columns [13]. The size of each square in the chessboard was 4 × 4 cm. To assure the accuracy of data acquisition, a reprojection error was calculated. The sampling frequency was 30 Hz in tablets.

### Procedure

All participants were asked to stand behind the tooth extraction simulator and execute tooth extraction toward the right side and left side referred as condition 1 and 2, respectively. In each condition, each participant was asked to keep tilting the tooth for fifty seconds with the right hand. All participants used their right hand to execute the tooth extraction.

### Data analysis

Mediapipe pose model was used to extract human joint locations from each video frame in each calibrated camera view. The extracted joint locations were triangulated to reconstruct 3D data [14]. The reconstructed data was filtered using lowpass Butterworth filter with the order of 4. The cutoff frequency was decided using the Residual method [15]. Joint angles of interests (see Table 1) were calculated using inverse trigonometry.

**Table 1:** Joint angles of interest and their definition

Joint angles of interests	Definition
Left shoulder	Angle between left upper arm and left side of trunk
Right shoulder	Angle between right upper arm and right side of trunk
Forward lean	Angle between vertical line and middle of trunk on sagittal plane
Side lean	Angle between vertical line and middle of trunk on frontal plane
Forward neck lean	Angle between vertical line and middle of neck on sagittal plane
Side neck lean	Angle between vertical line and middle of neck on frontal plane
Trunk rotation	Angle between left-right shoulder line and left-right hip line on transverse plane

### Statistics

The joint angles of interest were averaged over time and grouped based on the categories. T-tests with <0.05 *p*-value were used to compare dentistry students and young dentists with experienced dentists. All the data analysis was done by Python.

## Result

### Reprojection error

The reprojection error of the front view camera was 0.036 pixels. The reprojection error of the front view camera was 0.034 pixels.

### Mean joint angles

Table 2 shows mean and standard deviation of mean joint angles of interests. Neck side lean in group 2 vs 3 in both conditions were not significantly different although the mean difference was more than 10 degrees. Only right shoulder angles were significantly different in both comparisons and conditions.

**Table 2:** Difference and *p*-value of mean joint angles of interests among different groups

Joint angle	Group 1 (mean ± sd)	Group 2 (mean ± sd)	Group 3 (mean ± sd)	Group 1 vs 3 (mean diff [*p*-value])	Group 2 vs 3 (mean diff [*p*-value])
Condition 1					
Right shoulder	33.6 ± 3.0	34.8 ± 5.0	25.3 ± 0.1	8.4 [0.001]	9.6 [0.007]
Left shoulder	34.8 ± 6.2	36.7 ± 7.1	37.1 ± 0.3	−2.3 [0.5]	−0.4 [0.9]
Forward lean	4.4 ± 1.7	4.1 ± 2.9	2.7 ± 0.5	1.7 [0.1]	1.5 [0.3]
Side lean	4.4 ± 2.9	2.5 ± 2.8	2.9 ± 1.6	1.4 [0.4]	−0.5 [0.1]
Neck forward lean	39.9 ± 5.1	40.0 ± 13.2	38.3 ± 5.3	1.5 [0.8]	1.7 [0.2]
Neck side lean	5.3 ± 4.8	11.9 ± 8.4	1.6 ± 1.3	3.7 [0.2]	10.3 [0.09]
Trunk rotation	7.0 ± 5.0	3.8 ± 2.3	5.9 ± 3.1	1.0 [0.8]	−2.15 [0.5]
Condition 2					
Right shoulder	35.9 ± 4.8	35.6 ± 6.6	24.9 ± 1.9	11.0 [0.02]	10.8 [0.01]
Left shoulder	33.2 ± 3.9	33.8 ± 3.5	33.4 ± 0.2	−0.2 [0.9]	0.4 [0.8]
Forward lean	3.2 ± 0.9	5.1 ± 4.8	1.6 ± 0.3	1.7 [0.04]	3.5 [0.1]
Side lean	2.8 ± 3.5	2.0 ± 1.2	2.9 ± 1.2	−0.1 [1.0]	−0.9 [0.5]
Neck forward lean	45.8 ± 7.0	43.6 ± 15.4	40.1 ± 1.4	5.6 [0.2]	3.4 [0.6]
Neck side lean	25.9 ± 7.3	20.4 ± 13.9	7.5 ± 0.6	18.4 [0.01]	12.8 [0.07]
Trunk rotation	9.3 ± 6.1	8.0 ± 4.0	6.1 ± 3.3	3.1 [0.5]	1.9 [0.6]

### Standard deviation of joint angles

Table 3 shows mean and standard deviation of standard deviation of joint angles of interests. In condition 1, both sides of shoulder angles were significantly different in both comparisons although it was not the case in condition 2. Neck forward lean was significantly different in group 2–3 comparison in condition 2. All the significant differences were positive values, which means that the standard deviation of joint angles was smaller in group 3 than in comparing groups.

**Table 3:** Difference and p-value of standard deviation of joint angles of interests among different groups

Joint	Group 1 (mean ± sd)	Group 2 (mean ± sd)	Group 3 (mean ± sd)	Group 1 vs 3 (mean diff [p-value])	Group 2 vs 3 (mean diff [*p*-value])
Condition 1					
Right shoulder	2.9 ± 0.6	1.9 ± 0.6	1.1 ± 0.1	1.8 [0.006]	0.8 [0.02]
Left shoulder	1.4 ± 0.2	1.7 ± 0.7	0.8 ± 0.0	0.6 [0.02]	0.9 [0.03]
Forward lean	1.0 ± 0.4	1.0 ± 0.6	0.6 ± 0.0	0.4 [0.1]	0.4 [0.1]
Side lean	1.4 ± 1.2	1.1 ± 0.7	0.6 ± 0.0	0.8 [0.3]	0.5 [0.1]
Neck forward lean	2.2 ± 0.9	3.0 ± 1.5	2.0 ± 0.2	0.2 [0.7]	1.0 [0.2]
Neck side lean	5.0 ± 2.9	4.4 ± 3.0	1.9 ± 0.1	3.1 [0.1]	2.5 [0.09]
Trunk rotation	1.3 ± 0.4	1.2 ± 0.4	1.0 ± 0.2	0.3 [0.3]	0.1 [0.5]
Condition 2					
Right shoulder	2.3 ± 0.9	1.9 ± 1.2	1.2 ± 0.1	1.1 [0.09]	0.7 [0.2]
Left shoulder	1.4 ± 0.3	1.0 ± 0.5	0.9 ± 0.2	0.5 [0.1]	0.1 [0.6]
Forward lean	1.3 ± 0.9	0.7 ± 0.2	0.7 ± 0.1	0.6 [0.2]	−0.0 [1.0]
Side lean	1.0 ± 0.8	0.7 ± 0.2	0.8 ± 0.3	0.1 [0.8]	−0.2 [0.5]
Neck forward lean	2.7 ± 1.8	2.8 ± 1.1	1.4 ± 0.4	1.3 [0.2]	1.4 [0.04]
Neck side lean	3.7 ± 2.2	3.5 ± 1.9	1.6 ± 0.1	2.1 [0.2]	1.9 [0.06]
Trunk rotation	1.4 ± 0.3	1.1 ± 0.4	1.0 ± 0.4	0.4 [0.4]	0.1 [0.7]

## Discussion

The mean joint angles’ difference would indicate how kinematically the dentistry students and young dentists differ from experienced dentists. The significant difference in mean right shoulder angles would mean that the experienced dentists tighten their armpit more than the dentistry students and young dentists. Since all participants simulated the tooth extraction on their right hand, the difference would have been more pronounced. That would be why the mean left shoulder angle was not significantly different. A previous study [11] defined a poor posture in a shoulder angle as more than 30 degrees. Both young dentists and students were categorized as having poor posture although the experienced dentists were not in this evaluation method. In that sense, pose estimation could capture the posture and can be used as an evaluation tool. However, another study mentioned that the ideal shoulder angle should be less than 10 degrees in a sitting position [16]. This study asked participants to operate in a standing position; therefore, the shoulder angle may have been wider than the degrees the study suggested. The neck side lean was not significantly different between group 1–2 and group 3 although the difference was relatively bigger than other joint angles’ difference. It would be due to small sample sizes. Also, standard deviation of the joint angles in neck forward and side lean was around 8–15 degrees in young dentists. This large standard deviation would mean that the young dentists’ skills vary a lot, which can be one reason the difference was not significant.

The mean standard deviation of joint angles would indicate how stably the participants executed the tooth extraction. The mean standard deviation of the right shoulder angle in experienced dentists was significantly lower than dentistry students and young dentists except for group 2–3 difference in condition 2. This would indicate that experienced dentists stabilized their right arm more than dentistry students and young dentists except for that non-significant case. Considering the significant difference in the mean right shoulder angle, it would be important to reduce the right shoulder angle to stabilize the right arm during the tooth extraction.

In a previous study, many dentists flexed their necks more than 30 degrees for 85% of the 4-hour operation and abducted their shoulders more than 30 degrees for more than 50% of the 4-h operation [17]. A previous study mentioned that prolonged shoulder abduction and neck flexion would lead to undesired loads to the splenius and trapezius muscles, which can cause neck and/or shoulder musculoskeletal injuries [18, 19]. To investigate postural muscle activities, electromyography (EMG) is often used and considered to be a valid measurement [20]. One EMG found that the 40 degrees of shoulder abduction leads to more loads on shoulder muscles compared to the 20 degrees [21]. Another EMG study revealed that 46 degrees of neck flexion caused 45% of maximum neck extension momentum, although 23 degrees of neck flexion showed 32% [22]. The results from those EMG studies would indicate that more shoulder abduction and neck flexion would cause unnecessary muscle loads, which may cause musculoskeletal disorders. This study only investigated kinematic variables, but the methodology has the huge potential to help researchers and practitioners detect higher shoulder abduction and neck flexion, which would cause musculoskeletal injuries.

This study invited only twelve participants, including two experienced dentists. Therefore, this study may not be able to provide statistically powerful results as well as proper average measurements of experienced dentists’ performance. However, this study’s focus is a unique approach to measuring dentists’ kinematic performance during tooth extraction. The future study will include more participants, especially experienced dentists. Further, this study only measured the dentists’ performance for fifty seconds, which does not account for the cumulative loads over a long time. In the future, this study will be extended to measuring dentists’ kinematics in real operations, which should take more than fifty seconds. This study’s approach is more feasible to do it than other motion capture approaches since it only requires video recordings to capture motions.

## Conclusion

The right shoulder angle was a main difference between the experienced dentists and the other groups. Other joint angles, especially neck forward and side lean angles, showed some differences, but it was not statistically significant with large standard deviations. Considering the large standard deviation, the young dentists’ group can be further sub-grouped based on their skills. A sample size was small in this study; therefore, further study with more participants would be needed to draw more concrete conclusions. Despite that, this study showed that pose estimation would be useful for kinematic analysis of dentists during the tooth extraction. Indeed, many practitioners struggle with musculoskeletal injuries due to high shoulder abduction and neck flexion. To avoid the problem at an early stage, young dentists and students should learn and gain the proper posture since it would be difficult to adapt the posture in the later period. Therefore, this unique methodology would contain a great possibility in this field and change the way to educate and evaluate dentistry students and young dentists in tooth extraction.

## Data Availability

The data, mainly videos, includes participants’ privacy; therefore, it cannot be shared openly.
