# Correction: Posture analysis during tooth extraction

**DOI:** 10.1038/s41405-025-00313-z

**Published:** 2025-02-25

**Authors:** Takashi Fukushima, Keisuke Sugahara, Kazuhiro Ito, Masahide Koyachi, Akihiro Nishiyama, Chihiro Kurihara, Shintaro Nakajima, Satoru Matsunaga, Akira Katakura

**Affiliations:** 1 Technical University of Munich, Chair of Performance Analysis and Sports Informatics, Georg-Brauchle-Ring 60/62, 80992 München, Germany; 2 Tokyo Gakugei University, Education Incubation Center, 4-1-1 Nukuikitamachi, Koganei, Tokyo 184-8501 Japan; 3 Tokyo Dental College, Department of Oral Pathobiological Science and Surgery, Tokyo Dental College, Tokyo, Japan 2-9-18, Kanda Misaki-cho, Chiyoda-ku, Tokyo Japan; 4 Tokyo Dental College, Department of Anatomy, Tokyo Dental College 2-9-18, Kanda Misaki-cho, Chiyoda-ku, Tokyo 101-0061 Japan

**Keywords:** Dental clinical teaching, Extended skills training in dentistry

Correction to: *BDJ Open* 10.1038/s41405-025-00311-1, published online 17 February 2025

In this article the author’s name Shintaro Nakajima was incorrectly written as Shintaro Nakajim. The original article has been corrected.

